# Clinical Findings in Migrants With Asymptomatic *Plasmodium* Infections

**DOI:** 10.1093/ofid/ofaf525

**Published:** 2025-09-02

**Authors:** Isabelle Eliasson, Katja Wyss, Rebecca Tafesse Bogale, Sofia Forsblom, Emil Lindquist, Caroline Rönnberg, Magnus Hansson, Soheir Beshara, Irene Nordling, Olof Hertting, Andreas Wångdahl, Anna Färnert

**Affiliations:** Division of Infectious Diseases, Department of Medicine Solna and Centre for Molecular Medicine, Karolinska Institutet, Stockholm, Sweden; Department of Infectious Diseases, Karolinska University Hospital, Stockholm, Sweden; Division of Infectious Diseases, Department of Medicine Solna and Centre for Molecular Medicine, Karolinska Institutet, Stockholm, Sweden; Department of Infectious Diseases, Karolinska University Hospital, Stockholm, Sweden; Division of Infectious Diseases, Department of Medicine Solna and Centre for Molecular Medicine, Karolinska Institutet, Stockholm, Sweden; Division of Infectious Diseases, Department of Medicine Solna and Centre for Molecular Medicine, Karolinska Institutet, Stockholm, Sweden; Department of Infectious Diseases, Karolinska University Hospital, Stockholm, Sweden; Division of Infectious Diseases, Department of Medicine Solna and Centre for Molecular Medicine, Karolinska Institutet, Stockholm, Sweden; Division of Infectious Diseases, Department of Medicine Solna and Centre for Molecular Medicine, Karolinska Institutet, Stockholm, Sweden; Department of Microbiology, Unit of Parasitology, Public Health Agency of Sweden, Solna, Sweden; Department of Clinical Chemistry, Karolinska University Laboratory, Karolinska University Hospital, Huddinge, Sweden; Department of Laboratory Medicine, Karolinska Institutet, Stockholm, Sweden; Department of Molecular Medicine and Surgery, Karolinska Institutet, Stockholm, Sweden; Department of Infectious Diseases, Karolinska University Hospital, Stockholm, Sweden; Paediatric Infectious Diseases, Astrid Lindgren Children's Hospital, Karolinska University Hospital, Stockholm, Sweden; Department of Women's and Children's Health, Karolinska Institutet, Stockholm, Sweden; Division of Infectious Diseases, Department of Medicine Solna and Centre for Molecular Medicine, Karolinska Institutet, Stockholm, Sweden; Department of Infectious Diseases, Västmanland Hospital, Västerås, Sweden; Life Sciences, Burnet Institute, Melbourne, Victoria, Australia; Division of Infectious Diseases, Department of Medicine Solna and Centre for Molecular Medicine, Karolinska Institutet, Stockholm, Sweden; Department of Infectious Diseases, Karolinska University Hospital, Stockholm, Sweden

## Abstract

**Background:**

Migrants from malaria-endemic areas may have asymptomatic parasitemia that persists after relocating to nonendemic countries. Recommendations on malaria screening and treatment of asymptomatic infections in migrants are lacking. The aim of this study was to explore the clinical features of subclinical blood-stage *Plasmodium* infections in migrants, to inform screening and management strategies.

**Methods:**

A retrospective observational study was performed to evaluate clinical data from medical records of asymptomatic sub-Saharan African migrants identified with parasitic infection within a screening study in Stockholm, Sweden. Clinical data from hospital outpatient visits were compared between malaria polymerase chain reaction (PCR)–positive and PCR-negative individuals, the latter assessed for schistosomiasis and/or strongyloidiasis.

**Results:**

Clinical features and chemistry tests from 65 *Plasmodium* PCR-positive individuals were compared with data from 54 PCR-negative individuals. Study participants with *Plasmodium* infection had a higher proportion of anemia (21.1% vs 6.1%, *P* = .048), elevated erythrocyte sedimentation rate (ESR) (58.1% vs 25.0%, *P* = .008), raised plasma/serum immunoglobulin M (30.5% vs 10.5%, *P* = .030), and splenomegaly (25.4% vs 2.5%, *P* = .002). After antimalarial treatment, splenomegaly and laboratory parameters improved in *Plasmodium*-infected individuals.

**Conclusions:**

Migrants with subclinical *Plasmodium* infection have a high proportion of splenomegaly and abnormal laboratory findings, such as anemia and elevated ESR. Screening and treatment of subclinical malaria infections could prevent adverse outcomes and should be considered both in endemic and nonendemic settings.

## Teaser

Asymptomatic Plasmodium infections are prevalent in malaria endemic areas and may persist in migrants from sub-Saharan Africa, with potentially negative health effects such as splenomegaly, anemia and signs of inflammation. Plasmodium infection should be considered in patients presenting with these findings, and interventions such as malaria screening strategies could lead to early detection and improve health in risk populations.

## Graphical Abstract

**Graphical Abstract:**
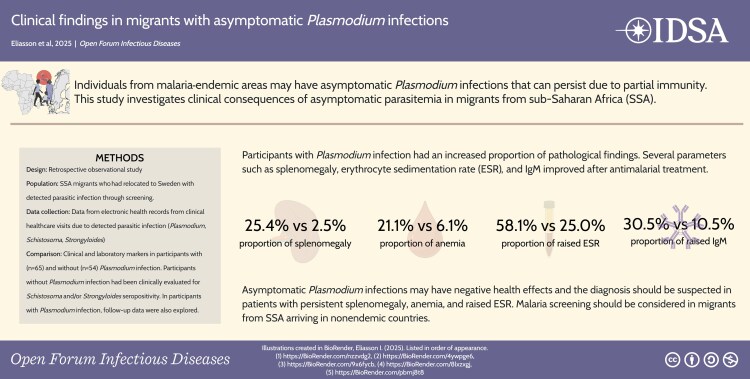
This graphical abstract is also available at Tidbit: https://tidbitapp.io/tidbits/clinical-findings-in-migrants-with-asymptomatic-plasmodium-infections?utm_campaign=tidbitlinkshare&amp;utm_source=ITP

Sub-Saharan Africa (SSA) has the highest global burden of malaria, accounting for 94% of the estimated 263 million cases and 95% of nearly 600 000 deaths in 2023 [1]. The clinical presentation of malaria infection varies, depending on previous exposure and preexisting malaria immunity. Individuals with limited or no previous exposure to the *Plasmodium* parasite are highly likely to develop clinical and potentially severe malaria. In contrast, those experiencing repeated exposure gradually develop immunity that protects from severe disease, and eventually against febrile malaria [2]. Nonetheless, this immunity is not sterilizing and while it provides partial protection from symptomatic malaria episodes, asymptomatic low-density infections with blood-stage malaria parasites are common across SSA [2].

International migration from Africa has increased [3]. Migration is associated with different health risks and migrants may come from areas with high burden of infectious diseases. Migrants from malaria-endemic countries may be asymptomatic carriers of malaria parasites, and these infections may persist after relocating to nonendemic areas such as Europe. A subset of these migrants will develop clinical malaria, generally leading to detection and treatment within the healthcare system. However, most subclinical infections are likely to remain asymptomatic and go undiagnosed, and even missed as causes of, for example, anemia. Currently, although screening for infections such as human immunodeficiency virus (HIV), hepatitis, and tuberculosis is recommended [4], no consensus has yet been reached on malaria screening in migrants arriving in Europe.

Within a parasite screening study in Sweden, we recently demonstrated that 10.4% of apparently asymptomatic SSA migrants screened at Migrant Health Assessment Units had blood-stage *Plasmodium* infections detected by real-time polymerase chain reaction (qPCR) [5]. In migrants reporting Uganda as last African country of residency, PCR positivity was 22.3% in adults and 35.8% in children [5]. A recent meta-analysis including 23 studies showed an overall 8% malaria PCR positivity in SSA migrants [6].

These findings raise the question whether malaria screening should be offered to SSA migrants; however, evident clinical implications are needed to guide intervention strategies. In endemic areas, asymptomatic *Plasmodium* infections are well-known to contribute to anemia and pregnancy-related complications such as low birth weight and maternal anemia [7, 8]. Associations with impaired school performance, bacteremia, Burkitt lymphoma, and increase in all-cause mortality have also been described [7, 9]. Furthermore, persistent low-density parasitemia may lead to hyperreactive malarial splenomegaly (HMS, earlier tropical splenomegaly syndrome) [10]. However, no comprehensive assessments have been performed in migrant populations. The aim of this study was to assess the clinical effects of asymptomatic *Plasmodium* infections in SSA migrants, in order to inform screening strategies and case management.

## METHODS

### Study Design and Study Population

This was a retrospective observational study comparing clinical data from individuals with and without *Plasmodium* infection participating in an ongoing parasite screening study in Sweden (ClinicalTrials.gov identifier NCT05086887, start date 15 April 2019) [5]. The study participants were retrieved from a cohort of 925 adults and children from SSA who had relocated to Sweden and were included mainly at the routine migrant health assessment offered to newly arrived migrants, as well as through announcements, letter invitations, or at latent tuberculosis visits at the Infectious Diseases Outpatient Clinic at Karolinska University Hospital. Hence, study participants were not actively seeking healthcare for fever or other symptoms. Participants screened before 28 July 2023 were included.

Participants found to be PCR positive for malaria parasites and/or seropositive for *Schistosoma* and/or *Strongyloides* in the screening study were referred to the Departments of Infectious Diseases or Pediatrics at Karolinska University Hospital for medical assessment and treatment. These parasite-positive individuals who underwent clinical assessment(s) for parasitic infection detected through the screening were then selected for this retrospective study. Individuals without parasitic infection were not included in this retrospective analysis, since they had not been referred for a clinical assessment.

### Data Collection

Clinical data were retrieved from electronic health records from the medical assessments for parasitic infections, as well as from prior medical visits. Follow-up data from follow-up visits after treatment, related to parasitic infection, were also retrieved for malaria PCR-positive participants (until 28 July 2023). Data included reported complaints, physical examination, laboratory, and radiology results, including radiology reports within 3 months of the initial medical assessment for parasitic infection.

### Definitions

Hepatomegaly and splenomegaly were defined by corresponding palpable or radiological findings. For the assessment of HMS, the major diagnostic criteria proposed by Fakunle [11] were used, and for assessment of early HMS, the criteria proposed by Bisoffi et al was used [12] (Supplementary Table 1). In terms of the HMS criteria, gross splenomegaly was defined as either a palpable spleen >10 cm below the costal margin [11] or a spleen length ≥16 cm measured by radiological examination [13]. Improvement of splenomegaly was defined as either a reduction in spleen size (by radiological measurement) or the absence of a palpable spleen upon the repeated physical examination. For laboratory parameters, age- and sex-adjusted reference ranges of the Karolinska University Laboratory were used (Supplementary Tables 2 and 3).

### Laboratory Analyses

Detection of malaria parasites within the screening study was performed by Carestart Pf/PAN (HRP2/pLDH) rapid diagnostic test (RDT) and species-specific qPCR [5], and additional diagnostics in the clinic included microscopy and qPCR. Serological screening for schistosomiasis and strongyloidiasis was later added to the original screening study and was performed on frozen plasma using the Bordier *Strongyloides ratti* immunoglobulin G (IgG) enzyme-linked immunosorbent assay (ELISA) and *Schistosoma mansoni* IgG ELISA kits. A limited number of study participants were already clinically screened for schistosomiasis and/or strongyloidiasis; in these cases, the clinical test results already determined by immunofluorescence and/or ELISA were used. Blood chemistry as well as data on other infections screened as part of the Migrant Health Assessment Program (HIV, hepatitis B/C, and tuberculosis) were retrieved from the medical records. To complement missing data, immunoglobulin M (IgM) and IgG quantification was performed by Karolinska University Laboratory through turbidimetry on frozen plasma samples. Other clinical chemistry analyses could not be performed on frozen samples. Moreover, IgG responses to *Plasmodium falciparum* crude schizont extract were analyzed by ELISA (modified protocol [14]).

### Data Analysis and Statistical Methods

Clinical data were compared between study participants with and without *Plasmodium* infection, the latter being clinically evaluated for *Schistosoma* and/or *Strongyloides* infection. In *Plasmodium*-infected individuals, clinical data were also compared between the initial clinical visit for *Plasmodium* infection and the follow-up visits after treatment.

Statistical analyses were performed in R 4.3.3 and GraphPad Prism 10.2 software. Fisher exact test was used to test associations between categorical variables. For continuous variables, the Mann-Whitney *U* test was used for independent observations and the Wilcoxon signed-rank test for paired data. Statistical significance was set at *P* < .05. Figures were created in GraphPad Prism 10.2.

### Ethical Considerations and Patient Consent Statement

All study participants, or their guardians, gave consent of participation after receiving oral and written information, with a translator when necessary. Study participants with detected parasitic infection were offered referral for clinical evaluation and treatment. The study was approved by the Swedish Ethical Review Authority (2019-00430 with amendments 2020-05351, 2022-01448-02, 2022-05557-02, and 2023-02210-02) and was performed in accordance with the Declaration of Helsinki.

## RESULTS

**Figure 1.**
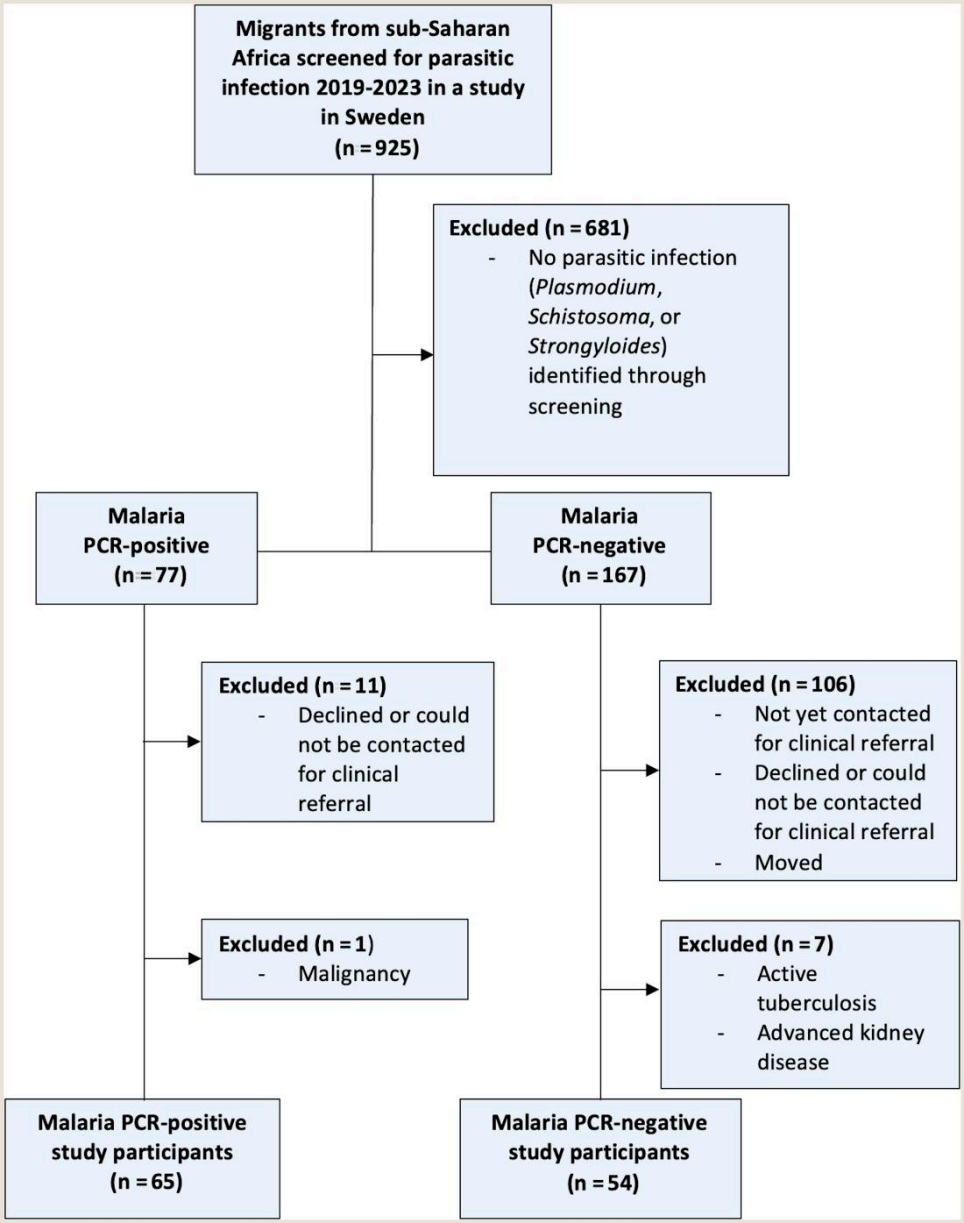
Flowchart of study inclusion. Malaria polymerase chain reaction (PCR)–negative study participants (n = 54) were all clinically assessed due to seropositivity for schistosomiasis and/or strongyloidiasis.

Among the SSA migrants with parasitic infections detected through the screening study, 65 individuals with *Plasmodium* infection and 54 individuals without *Plasmodium* infection had attended at least one clinical visit for parasitic infection and did not have considerable comorbidities (Figure 1). Among study participants with *Plasmodium* infection, 50.8% (33/65) were adults and 49.2% (32/65) were children; among those without *Plasmodium* infection, 85.2% (46/54) were adults and 14.8% (8/54) were children (Table 1). Study participants with *Plasmodium* infection were infected with *P falciparum* (33/65 [50.8%]), *Plasmodium ovale* (14/65 [21.5%]), *Plasmodium malariae* (9/65 [13.8%]), or mixed *Plasmodium* species (9/65 [13.8%]). In participants with *Plasmodium* infection, 19 of 61 (31.1%) were RDT positive in research testing at study inclusion, and 17 of 46 (37.0%) were microscopy positive in clinical testing. Quantitative parasite count was reported in 8 microscopy-positive participants with *P falciparum*, all with parasitemia ≤0.1%. Nine microscopy-positive participants had no reported parasite densities, but one participant had reported findings of *P malariae* gametocytes. Among participants with *Plasmodium* infection, 33.9% (21/62) were seropositive for *Schistosoma* and 21.0% (13/62) for *Strongyloides*, and 3 were not tested due to insufficient blood volume (Table 1).

**Table 1.** Characteristics of the Study Participants

Characteristic	Malaria PCR^+^ (n = 65)	Malaria PCR^−^ (n = 54)
Sex		
Male	26 (40.0)	28 (51.9)
Female	39 (60.0)	26 (48.1)
Age, y		
Median (range)	18 (2–59)	28 (11–53)
Children (<18 y)	32 (49.2)	8 (14.8)
Adults (≥18 y)	33 (50.8)	46 (85.2)
Region of birth^a^		
Central Africa	27 (41.5)	16 (29.6)
East Africa	35 (53.8)	30 (55.6)
Other African region	3 (4.6)	8 (14.8)
Last African region of residency^a^		
Central Africa	3 (4.6)	1 (1.9)
East Africa	59 (90.8)	37 (68.5)
Other African region	3 (4.6)	16 (29.6)
Days in Sweden at study inclusion		
Median (range)	66 (6–449)	155 (6–4805)
<100	34 (52.3)	21 (39.6)
100–299	12 (18.5)	12 (22.6)
300–999	19 (29.2)	10 (18.9)
≥1000	0 (0)	10 (18.9)
Missing, No.	0	1
Days from arrival in Sweden to clinical assessment		
Median (range)	135 (8–1408)	651.5 (69–5893)
Missing, No.	2	4
Malaria diagnostics, no./No. (%)		
RDT positive^b^	19/61 (31.1)	…
Microscopy positive^c^	17/46 (37.0)	…
PCR positive^b^	65/65 (100.0)	0/54 (0)
*Plasmodium* species (by PCR), no./No. (%)		
*P falciparum*	33/65 (50.8)	…
*P ovale*	14/65 (21.5)	…
*P malariae*	9/65 (13.8)	…
Mixed *Plasmodium* infection^d^	9/65 (13.8)	…
Coinfections, no./No. (%)		
*Schistosoma*, ELISA positive^e^	21/62 (33.9)	45/54 (83.3)
*Strongyloides*, ELISA positive^e^	13/62 (21.0)	17/54 (31.5)
HIV	0/60 (0)	1/43 (2.3)
Chronic hepatitis B	1/59 (1.7)	4/40 (10.0)
Active hepatitis C (RNA positive)	0/60 (0)	0/38 (0)
Latent tuberculosis	14/60 (23.3)	32/48 (66.7)^f^

Data are presented as No. (%) unless otherwise indicated. Some variables are presented as no./No. (%), where No. equals the number of study participants with available data for each variable. Sex, region of birth, last African region of residency, and days in Sweden are stated at the time of screening sampling; age and infection status are stated at the time of first clinical assessment for parasitic infection.

Abbreviations: ELISA, enzyme-linked immunosorbent assay; HIV, human immunodeficiency virus; PCR^+^, polymerase chain reaction positive; PCR^–^, polymerase chain reaction negative; RDT, rapid diagnostic test.

^a^According to the United Nations geoscheme.

^b^Research laboratory analysis.

^c^Clinical laboratory analysis.

^d^Two *P falciparum* + *P ovale* + *P malariae*, 4 *P falciparum* + *P ovale*, 2 *P falciparum* + *P malariae*, 1 *P ovale* + *P malariae*.

^e^Determined by Bordier *Strongyloides ratti* IgG ELISA kit and *Schistosoma mansoni* ELISA kit on research plasma or through clinical serological analysis.

^f^There was an overrepresentation of study inclusions at the latent tuberculosis clinic in subjects without *Plasmodium* infection.

### Clinical Presentation and Laboratory Findings

Study participants with *Plasmodium* infection had a higher proportion of anemia (21.1% vs 6.1%, *P* = .048), high erythrocyte sedimentation rate (ESR) (58.1% vs 25.0%, *P* = .008), raised plasma/serum IgM (30.5% vs 10.5%, *P* = .030), and splenomegaly (25.4% vs 2.5%, *P* = .002) as well as lower proportion of eosinophilia (8.3% vs 24.4%, *P* = .048), compared to those without *Plasmodium* infection (Table 2). There were no significant differences in reported complaints at the clinical visit or prior to the visit. Subanalyses on categorical data in children were not performed due to insufficient data, mainly due to the limited number of children without *Plasmodium* infection (Table 2).

**Table 2.** Clinical Presentation of Study Participants With and Without *Plasmodium* Infection in Adults and Children

Clinical Presentation	All			Adults, ≥18 y			Children, <18 y	
	Malaria PCR^+^ (n = 65)	Malaria PCR^−^ (n = 54)	*P* Value^a^	Malaria PCR^+^ (n = 33)	Malaria PCR^−^ (n = 46)	*P* Value^a^	Malaria PCR^+^ (n = 32)	Malaria PCR^−^ (n = 8)
Reported complaints, no./No. (%)								
Fever	9/65 (13.8)	3/54 (5.6)	.221	5/33 (15.2)	3/46 (6.5)	.268	4/32 (12.5)	0/8 (0)
Chills	5/65 (7.7)	1/54 (1.9)	.219	4/33 (12.1)	1/46 (2.2)	.155	1/32 (3.1)	0/8 (0)
Headache	11/65 (16.9)	9/54 (16.7)	1.000	8/33 (24.2)	8/46 (17.4)	.572	3/32 (9.4)	1/8 (12.5)
Body ache	3/65 (4.6)	4/54 (7.4)	.700	3/33 (9.1)	4/46 (8.7)	1.000	0/32 (0)	0/8 (0)
GI complaints	18/65 (27.7)	21/54 (38.9)	.240	12/33 (36.4)	17/46 (37.0)	1.000	6/32 (18.8)	4/8 (50.0)
Cough	2/65 (3.1)	1/54 (1.9)	1.000	1/33 (3.0)	1/46 (2.2)	1.000	1/32 (3.1)	0/8 (0)
Laboratory findings, no./No. (%)								
Anemia	12/57 (21.1)	3/49 (6.1)	.048	11/33 (33.3)	3/45 (6.7)	.006	1/24 (4.2)	0/4 (0)
Leukopenia^b^	15/57 (26.3)	11/49 (22.4)	.659	4/33 (12.1)	8/45 (17.8)	.544	11/24 (45.8)	3/4 (75.0)
Neutropenia	21/47 (44.7)	15/45 (33.3)	.292	9/31 (29.0)	14/43 (32.6)	.803	12/16 (75.0)	1/2 (50.0)
Eosinophilia	4/48 (8.3)	11/45 (24.4)	.048	4/31 (12.9)	9/43 (20.9)	.538	0/17 (0)	2/2 (100)
Thrombocytopenia	7/56 (12.5)	2/46 (4.3)	.180	6/32 (18.8)	2/42 (4.8)	.070	1/24 (4.2)	0/4 (0)
Elevated ESR	18/31 (58.1)	11/44 (25.0)	.008	15/27 (55.6)	10/42 (23.8)	.011	3/4 (75.0)	1/2 (50.0)
Hypoalbuminemia	10/31 (32.3)	5/32 (15.6)	.148	8/26 (30.8)	5/31 (16.1)	.220	2/5 (40.0)	0/1 (0)
Elevated IgM	18/59 (30.5)	4/38 (10.5)	.030	14/31 (45.2)	4/30 (13.3)	.011	4/28 (14.3)	0/8 (0)
Elevated IgG	47/59 (79.7)	25/38 (65.8)	.156	27/31 (87.1)	22/30 (73.3)	.211	20/28 (71.4)	3/8 (37.5)
Elevated LDH	18/29 (62.1)	13/30 (43.3)	.195	18/27 (66.7)	13/29 (44.8)	.116	0/2 (0)	0/1 (0)
Other findings, no./No. (%)								
Hepatomegaly	1/59 (1.7)	1/40 (2.5)	1.000	1/29 (3.4)	1/33 (3.0)	1.000	0/30 (0)	0/7 (0)
Splenomegaly	15/59 (25.4)	1/40 (2.5)	.002	11/29 (37.9)	1/33 (3.0)	.0007	4/30 (13.3)	0/7 (0)

Variables presented as no./No. (%), where No. equals the number of study participants with available data for each variable. *P* values were not determined in children, due to the limited number of PCR-negative children and the amount of missing data.

Abbreviations: ESR, erythrocyte sedimentation rate; GI, gastrointestinal; IgG, immunoglobulin G; IgM, immunoglobulin M; LDH, lactate dehydrogenase; PCR^+^, polymerase chain reaction positive; PCR^–^, polymerase chain reaction negative.

^a^Fisher exact test; PCR-positive and PCR-negative adults compared.

^b^No leukocytosis observed.

**Figure 2.**
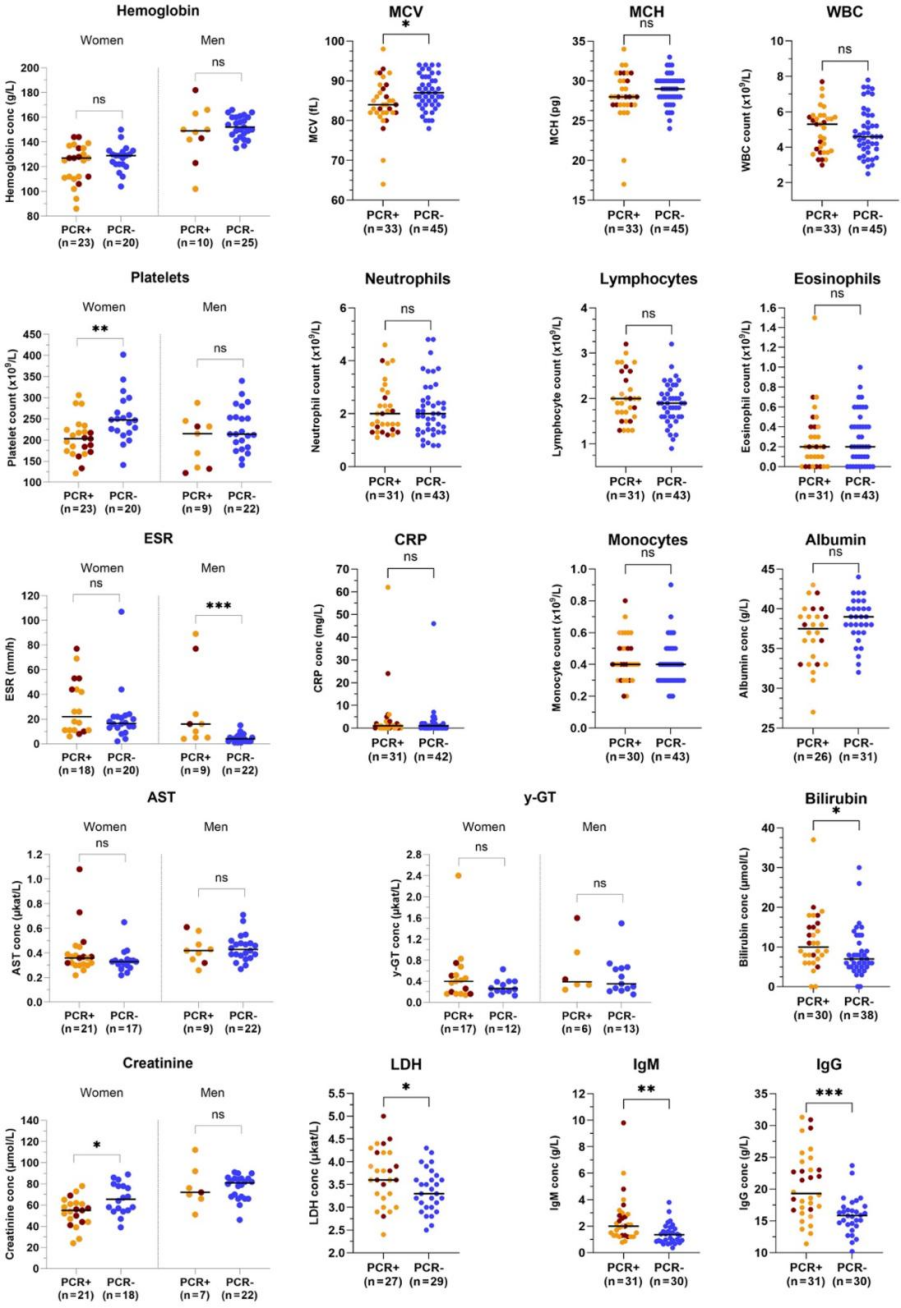
Laboratory parameters in adults with and without *Plasmodium* infection. Divided by sex when appropriate according to reference ranges. Data on gamma-glutamyl transferase in this graph only include adults 18–40 y of age, due to age-adjusted reference ranges. Available data for each parameter are shown. Study participants with *Plasmodium* infection (left) without splenomegaly or with unknown spleen size are labeled in light orange. Participants with *Plasmodium* infection and splenomegaly are labeled in dark red. Participants without *Plasmodium* infection (right) are labeled in blue. The median is represented by a black bar for both participants with and without *Plasmodium* infection. Statistical analysis with Mann-Whitney *U* test; not significant (ns) *P* ≥ .05, *P < .05, **P < .01, ***P < .001. Abbreviations: AST, aspartate aminotransferase; conc, concentration; CRP, C-reactive protein; ESR, erythrocyte sedimentation rate; IgG, immunoglobulin G; IgM, immunoglobulin M; LDH, lactate dehydrogenase; PCR+, malaria polymerase chain reaction positive; PCR–, malaria polymerase chain reaction negative; MCH, mean corpuscular hemoglobin; MCV, mean corpuscular volume; WBC, white blood cells; y-GT, gamma-glutamyl transferase.

Adults with *Plasmodium* infection had higher median plasma/serum levels of lactate dehydrogenase (LDH) (3.6 vs 3.3 µkat/L, *P* = .047), bilirubin (10.0 vs 7.0 µmol/L, *P* = .019), IgM (2.0 vs 1.35 g/L, *P* = .001), and IgG (19.3 vs 15.85 g/L, *P* = .0001) compared to participants without *Plasmodium* infection. Moreover, adults with *Plasmodium* infection had lower mean corpuscular volume (MCV) (84.0 vs 87.0 fL, *P* = .029) (Figure 2). There was also a lower median platelet count (203.0 vs 247.5 10^9^/L, *P* = .002) and plasma creatinine in women with *Plasmodium* infection (55.0 vs 65.5 μmol/L, *P* = .013). Additionally, men with *Plasmodium* infection had higher ESR (16.0 vs 4.0 mm/h, *P* = .0007) (Figure 2). Continuous data were not compared in children, due to age-adjusted reference ranges, which limited the number of participants in each group.

### Splenomegaly and *Plasmodium* Infection

Splenomegaly was more frequently observed in adults with *Plasmodium* infection (11/29 [37.9%]) compared to adults without *Plasmodium* infection (1/33 [3.0%], *P* = .0007). In children, splenomegaly was found in 4/30 (13.3%) with *Plasmodium* infection and in none of children without parasitemia (Table 2). Among the 15 study participants with splenomegaly and *Plasmodium* infection, splenomegaly was detected by radiology in 7 participants, 5 were described as having “palpable spleen” and/or “splenomegaly,” and 3 as having “suspected or probable splenomegaly” but without radiology. Spleen size (maximal longitudinal spleen length) by radiological measurement was described in 5 participants with splenomegaly. Two adults had a spleen size of 17 cm and 19 cm, respectively, and 3 children had a spleen size of 11 cm, 12 cm, and 12.5 cm, respectively. One participant with *Plasmodium* infection had “suspected” hepatomegaly. Among study participants without *Plasmodium* infection, one had “suspected hepatosplenomegaly” by palpation, but underwent no radiological examination.

Participants with *Plasmodium* infection and splenomegaly had a higher proportion of raised plasma/serum IgM (60.0% vs 18.4%, *P* = .006), and adults with splenomegaly had a higher proportion of neutropenia (60.0% vs 11.1%, *P* = .011) (Supplementary Table 4). Although exact spleen measurements were not described in all participants, 2 of 15 participants still fulfilled all major Fakunle criteria [11] and were considered to have HMS (Supplementary Table 5). In addition, 15 of 15 fulfilled the criteria of early HMS (Supplementary Table 5). No significant correlation of the infecting *Plasmodium* species and the occurrence of splenomegaly could be found. There was no association between splenomegaly and any other coinfection (Supplementary Table 4).

### Follow-up

**Figure 3.**
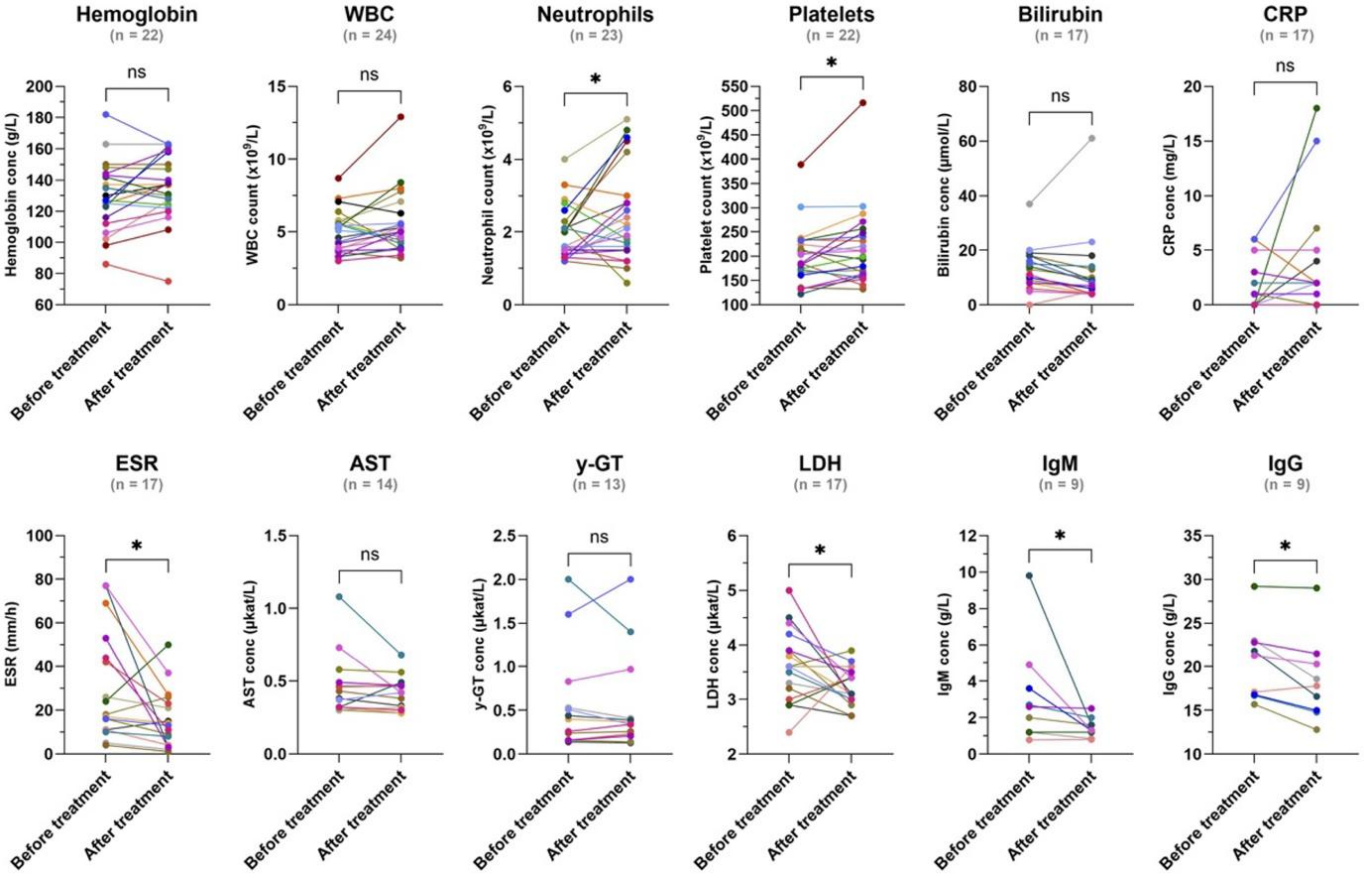
Laboratory findings at the first clinical visit (before treatment) vs the last follow-up after antimalarial treatment in *Plasmodium*-infected adults and children. Available data for each parameter are shown. Follow-up visits <1 mo from the first clinical visit were excluded. Statistical analysis with Wilcoxon matched-pairs signed-rank test; not significant (ns) *P* ≥ .05, *P < .05. Abbreviations: AST, aspartate aminotransferase; conc, concentration; CRP, C-reactive protein; ESR, erythrocyte sedimentation rate; IgG, immunoglobulin G; IgM, immunoglobulin M; LDH, lactate dehydrogenase; WBC, white blood cells; y-GT, gamma-glutamyl transferase.

In study participants with *Plasmodium* infection, the average time from the initial clinical visit for detected *Plasmodium* infection to the last recorded follow-up was 198 days (range, 6–1060 days). The late follow-ups were primarily related to medical assessments due to seropositivity identified in the later serological screening for *Strongyloides* and *Schistosoma* (Table 1). Comparing data from the initial clinical visits with follow-up visits >1 month after receiving antimalarial treatment, participants improved in neutrophil count (*P* = .049), platelet count (*P* = .049), ESR (*P* = .019), LDH (*P* = .043), IgM (*P* = .016), and IgG (*P* = .012) (Figure 3, Supplementary Figures 1 and 2). Splenomegaly in *Plasmodium*-infected participants resolved completely in 8 of 15 and partially in 3 of 15, while 4 of 15 participants did not have any follow-up visit examining spleen size.

## DISCUSSION

This retrospective observational study assessed clinical features in SSA migrants who were found positive for *Plasmodium* infection in a PCR-based screening study in Sweden. Clinical findings of splenomegaly and laboratory abnormalities such as high ESR, hypergammaglobulinemia, and anemia were prominent in study participants with *Plasmodium* infection. After antimalarial treatment, participants with *Plasmodium* infection had significant improvement of multiple laboratory parameters such as ESR and immunoglobulin levels, as well as partial or complete recovery of splenomegaly. These findings suggest that asymptomatic *Plasmodium* infections in SSA migrants have hidden clinical consequences, which may persist unless antimalarial treatment is provided.

Current knowledge on the pathogenesis and clinical consequences of asymptomatic *Plasmodium* infections is limited. However, previous research has suggested a strong link between asymptomatic *Plasmodium* infections and anemia as well as pregnancy-related complications [7, 8]. Here, we detected an effect also on other parameters in study participants with *Plasmodium* infection, where especially splenomegaly and high ESR stood out as potential indicators of subclinical infections.

In our results, raised ESR was highly prevalent in participants with *Plasmodium* infection. This finding has not previously been well described, although we found one report with findings of raised ESR in asymptomatic *Plasmodium* infections [15]. There are, however, further reports of an increase of other inflammatory markers during asymptomatic *Plasmodium* infection, such as tumor necrosis factor (TNF)–α, interleukin 6 (IL-6), and low-grade C-reactive protein (CRP) elevation [16–19]. However, one study did not see a clear increase of TNF and IL-6 in asymptomatic parasitemia but, as expected, observed higher levels of TNF and IL-6 in individuals with acute malaria compared to those with asymptomatic infection [20]. Our findings of elevated ESR may further support that asymptomatic *Plasmodium* infections are associated with inflammation. This inflammation is more likely chronic than acute, since CRP was not raised. However, raised ESR may also relate to hypergammaglobulinemia.

Splenomegaly has been reported in SSA migrants, and *Plasmodium* infections have been proposed as a possible cause; however, infections have not been systematically assessed [21, 22]. Historically, splenomegaly rates were used as malaria endemicity indicators, before being replaced by parasite prevalence [23]. Splenomegaly may occur in acute malaria, but also in subclinical infections where HMS may develop. Continuous *Plasmodium* antigen stimulation is thought to trigger an intense polyclonal B-cell response resulting in high IgM levels and splenic immune complex deposition [24]. HMS is associated with raised serum IgM and with pancytopenia indicating hypersplenism [11]. Indeed, study participants with splenomegaly and *Plasmodium* infection had an increased proportion of both high IgM levels and neutropenia, suggesting that at least part of the splenomegaly could be driven by the HMS mechanism. Although few participants fulfilled all major HMS criteria, 15 of 15 fulfilled the early HMS criteria. Early HMS is thought to predispose for fulminant disease [12, 25]. There may also be other mechanisms contributing to splenomegaly. Recent studies suggest that during asymptomatic parasitemia, the spleen may harbor a large proportion of the infecting *Plasmodium* parasite load [26] as well as uninfected red blood cells [27], potentially contributing to anemia and splenomegaly. Here, no significant increase in anemia was seen in participants with splenomegaly, suggesting that splenomegaly may not have been a major driver of anemia in our cohort and that there may be other important mechanisms of anemia that are present both in study participants with and without splenomegaly. It should be noted that not everyone was systematically assessed for other causes of splenomegaly, including for sickle cell disease. However, no associations between splenomegaly and *Schistosoma* or *Strongyloides* infection were found.

Anemia in asymptomatic *Plasmodium* infection is likely multifactorial, and markers of hemolysis, impaired erythropoiesis, inflammation, and altered iron metabolism have previously been observed [18, 28]. Anemia was more frequent in participants with *Plasmodium* infection; however, the proportion of anemia in children was surprisingly low. Additional laboratory findings of low MCV and increased serum bilirubin indicate that hemolysis and iron deficiency contributed to anemia. Another notable finding was the high proportion of participants with *Plasmodium* infection and raised plasma LDH. Although raised LDH could further relate to hemolysis, it could also represent damage of other tissues, which could in future studies be determined by measuring the levels of LDH isoenzymes. Furthermore, thrombocytopenia, a common feature in acute malaria, has also been observed in asymptomatic infections [29, 30]. In our results, the platelet count was lower in women with *Plasmodium* infection, but this correlation was not clear in men.

Whether there is causality between the pathological clinical findings observed in study participants and asymptomatic *Plasmodium* infection cannot be determined through this study alone. Nonetheless, study participants with *Plasmodium* infections did have significantly more evident pathology, which then improved after antimalarial treatment, suggesting an association. It should however be noted that some clinical parameters may be influenced by conditions before arrival in Sweden such as living situation and nutritional status, and that the time between arrival and clinical assessment must be considered. This time was overall shorter in participants with *Plasmodium* infection; however, it is unlikely that these factors would account for all pathological findings. The time between arrival in Sweden and diagnosis was in general still relatively long. In participants with *Plasmodium* infection, around half of study participants had resided in Sweden for >100 days at the time of screening detection.

Our findings add to previous evidence of negative effects associated with asymptomatic *Plasmodium* infections. Here we describe the clinical implications of asymptomatic parasitemia not only in children, but also in adults. A large proportion of these *Plasmodium* infections were persistent, since they were detected after several months and even years in Sweden. Although the exact duration of asymptomatic parasitemia is not established, there are case reports of *Plasmodium* infections persisting years after exposure [31], which raises the concern of long-term infections and health complications. Indeed, several study participants had potential signs of low-grade chronic inflammation, which could increase the risk of type 2 diabetes and cardiovascular disease [32]. A potential role of malaria-driven inflammation as a contributor of hypertension development has also been suggested [33]. Furthermore, HMS has been linked to several negative outcomes including a potential link to the development of splenic marginal zone lymphoma [34, 35].

Altogether, our findings suggest that asymptomatic *Plasmodium* infections are not without clinical consequences and should be prevented and treated. Apart from previously discussed concerns, subclinical placental *Plasmodium* infections during pregnancy may have serious consequences such as low birth weight, premature birth, and miscarriage [8]. In malaria-endemic areas, the World Health Organization recommends presumptive treatment for risk groups by providing seasonal or perennial malaria chemoprevention to children and intermittent preventive treatment to pregnant women [36]. However, further interventions to improve coverage and/or target asymptomatic parasitemia outside of these groups may be important. In nonendemic areas, active screening and treatment of malaria infections in migrants from endemic countries could have public health implications such as preventing potential transmission through local vectors or through blood transfusion, organ transplantation, and vertical transmission [37, 38], but could also, as shown here, have significant health benefits for the tested individual. Our findings may also help with raising awareness among clinicians treating patients of SSA origin that asymptomatic *Plasmodium* infections should be considered in patients with persistent anemia, high ESR, or splenomegaly. Early recognition of *Plasmodium* infection would help prevent prolonged investigations with sometimes potentially harmful interventions, as in cases where splenectomy has been performed due to HMS being mistaken for malignancy [39, 40].

### Strengths and Limitations

The main limitation of this study is the retrospective design without standardized data collection, resulting in missing data and risk of bias, especially in children. Participants with more pathological findings may have had more extensive evaluation. Moreover, clinical investigations were not available for individuals without detected parasitic infection in the screening cohort [5], as they were not referred for clinical assessment. Instead, study participants who had been evaluated for suspected *Schistosoma* and/or *Strongyloides* infection were used as a control group, which may lead to an underestimation of clinical effects. Demographic differences between the groups could affect results. Therefore, data were stratified and reference ranges were adjusted for age and sex. Furthermore, semi-immune individuals may have very low-density parasitemia, falling under the PCR detection limit; hence, some study participants in the malaria PCR-negative group could actually have had very low-density undetected parasitemia. It should be noted that there was no correction for multiple testing. This was decided due to the small sample size and to prevent significant findings being rejected as not significant. Therefore, the findings of this study should be confirmed in a larger systematic setting. A strength of this study is the nonendemic setting, making it possible to assess the impact of persistent low-density parasitemia in individuals included after some time in Sweden and without interfering cofactors such as reinfections in endemic areas. Moreover, the study was performed in a clinical context, making the results highly relevant for future guidelines.

### Future Research

Future prospective studies including a systematic clinical assessment at the time of screening could provide even more solid evidence. Furthermore, establishing the duration of *Plasmodium* infections is important to ensure optimal timing of new interventions. Moreover, improving the understanding of asymptomatic parasitemia also in endemic settings is important for guiding case management and preventive measures.

## CONCLUSIONS

Sub-Saharan African migrants with subclinical blood-stage *Plasmodium* infections may experience negative health effects despite being apparently asymptomatic. These findings indicate that detecting and treating asymptomatic parasitemia could improve the health of affected populations. Asymptomatic *Plasmodium* infection should be suspected in patients from malaria-endemic countries presenting with splenomegaly and/or laboratory findings such as anemia and elevated ESR. Future prospective systematic cohort studies could further elucidate the effects of asymptomatic parasitemia.

## Supplementary Material

ofaf525_Supplementary_Data

## Supplementary Data

Supplementary materials are available at *Open Forum Infectious Diseases* online. Consisting of data provided by the authors to benefit the reader, the posted materials are not copyedited and are the sole responsibility of the authors, so questions or comments should be addressed to the corresponding author.
