# Supplementary material for: Clinical Findings in Migrants With Asymptomatic Plasmodium Infections
Source: Open Forum Infect Dis. 2025 Sep 2;12(9):ofaf525. doi: 10.1093/ofid/ofaf525 (PMC12418173; doi:10.1093/ofid/ofaf525)
Supplement: ofaf525_Supplementary_Data [file ofaf525_supplementary_data.docx]

**Supplementary data**

**Supplementary table 1.** Complete diagnostic criteria for hyperreactive malarial splenomegaly (HMS) and early HMS (eHMS)

| **HMS criteria *(Fakunle, Clin Haematol, 1981)*** |  | **Early HMS criteria *(Bisoffi et al, Malar J, 2015)*** |
| --- | --- | --- |
| **Major diagnostic criteria** |  | - Anti-malarial antibody titre (IFAT- |
| - Gross splenomegaly |  | Biomérieux) > 1/160 |
| - Immunity to malaria |  | PLUS |
| - Serum IgM at least two standard deviations (SD) above the local mean |  | - Splenomegaly (echographic longitudinal |
|  |  | diameter ≥ 12 cm or palpable lower pole of the |
| - Clinical and immunological response to antimalarials |  | spleen) |
|  |  | AND/OR |
| **Minor diagnostic criteria** |  | - High IgM level (≥2.5 g/L) |
| - Hepatic sinusoidal lymphocytosis (HSL) |  | AND |
| - Normal cellular and humoral immune response to |  | - No other identified causes of splenomegaly or of |
| antigenic challenge |  | raised IgM, such as: schistosomiasis, HBV, HCV, HIV, |
| - Normal phytohaemagglutinination (PHA) response |  | brucellosis, leishmaniasis, autoimmune disease, |
| - Hypersplenism |  | cirrhosis, haemoglobinopathies, and other |
| - Lymphocytic proliferation |  | haematological conditions, such as leukemia, |
| - Occurrence in families |  | lymphoma, Waldenstrom disease. |

**Supplementary table 2.** Clinical laboratory tests, reference ranges in adults by sex and age

| **Laboratory examination** | **Reference range** |
| --- | --- |
| B-Hemoglobin, g/L |  |
| - Females | 117-153 |
| - Males | 134-170 |
| B-Hematocrit |  |
| - Females | ​0.35-0.46 |
| - Males | ​0.39-0.50 |
| B-MCV, fL | 82-98 |
| B-MCH, pg | 27-33 |
| B-Leukocyte count, cells x 10^9^/L | 3.5-8.8 |
| B-Neutrophils, cells x 10^9^/L | 1.6-5.9 |
| B-Lymphocytes, cells x 10^9^/L | ​1.1-3.5 |
| B-Monocytes, cells x 10^9^/L | 0.2-0.8​ |
| B-Eosinophils, cells x 10^9^/L | 0.0-0.5 |
| B-Basophils, cells x 10^9^/L | 0.0-0.1​ |
| B-Platelet count, cells x 10^9^/L |  |
| - Females | ​165-387 |
| - Males | ​145-348 |
| P-C-reactive protein (CRP), mg/L | <3 |
| B-Erythrocyte sedimentation rate (ESR), mm/h |  |
| - Females < 60 years | ​< 20 |
| - Females ≥ 60 years | ​<30 |
| - Males < 60 years | ​< 10 |
| - Males ≥ 60 years | <20 |
| P-Albumin, g/L |  |
| - 18-39 years | ​36-48 |
| - ​40-69 years | ​36-45 |
| - ≥70 years | 34-45 |
| P/S-IgM, g/L | ​0.27-2.10 |
| P/S-IgG, g/L | ​6.70-14.5 |
| Creatinine, micromole/L |  |
| - Females | <90 |
| - Males | <100 |
| P-Aspartateaminotransferase (AST), microkat/L |  |
| - Females | <0.61 |
| - Males | <0.76 |
| P-Alanineaminotransferase (ALT), microkat/L |  |
| - Females | <0.76 |
| - Males | <1.1 |
| P-Gamma-glutamyltransferase (y-GT), mikrokat/L |  |
| - Females 18-40 years | <0.76 |
| - Females ≥41 years | <1.4 |
| - Males 18-40 years | <1.3 |
| - Males ≥41 years | <2.0 |
| P-Bilirubin, micromole/L | <26 |
| P-Lactatedehydrogenase (LDH), microkat/L |  |
| - 18-70 years | ≤3.5 |
| - ​≥71 years | ≤4.3 |

**Supplementary table 3.** Clinical laboratory tests, reference ranges in children by sex and age

| **Laboratory examination** | **Reference range** |
| --- | --- |
| B-Hemoglobin, g/L |  |
| - <2 days | ​150-240 |
| - 2-6 days | ​140-220 |
| - 7-21 days | ​130-200 |
| - 22-28 days | 100-180 |
| - 1-3 months | 100-160 |
| - 4-12 months | ​100-140 |
| - 1-6 years | ​100-150 |
| - 7-10 years | 105-150 |
| - 11-17 years | 110-160​ |
| B-hematocrit |  |
| - <2 days | 0.45-0.59 |
| - 2-6 days | 0.57-0.70 |
| - 7-14 days | 0.47-0.59 |
| - 15-28 days | 0.38-0.52 |
| - 1-12 months | 0.34-0.42 |
| - 1-10 years | 0.37-0.41 |
| - 11-17 years | 0.38-0.46 |
| B-MCV, fL |  |
| - <2 days | 104-116 |
| - 2-6 days | 100-120 |
| - 7-21 days | 97-110 |
| - 22-28 days | 93-108 |
| - 1-3 months | 89-100 |
| - 4 months-17 years | 76-94 |
| B-MCH, pg |  |
| - <2 days | 30-40 |
| - 2-28 days | 30-36 |
| - 1-3 months | 28-35 |
| - 4-12 months | 25-32 |
| - 1-6 years | 24-30 |
| - 7-10 years | 26-30 |
| - 11-17 years | 27-31 |
| B-Leukocyte count, cells x 10^9^/L |  |
| - < 2 days | ​9.0-30.0 |
| - ​2-6 days | ​5.0-25.0 |
| - ​7-28 days | ​5.0-20.0 |
| - 1-3 months | ​6.0-18.0 |
| - 4 months-3 years | ​6.0-16.0 |
| - ​4-6 years | 5.0-15.0 |
| - ​7-17 years | ​5.0-13.0 |
| B-Neutrophils, cells x 10^9^/L |  |
| - < 2 days | ​5.0-25.5 |
| - ​ 2-6 days | 1.5-15.0 |
| - 7-28 days | ​1.0-11.0 |
| - ​ 1-4 months | 1.2-8.1 |
| - ​ 5-12 months | 1.2-7.2 |
| - ​ 1-2 years | 1.5-8.8 |
| - ​3-4 years | ​2.0-9.6 |
| - 5-6 years | ​1.5-8.9 |
| - 7-10 years | ​2.0-8.4 |
| - ​ 11-17 years | ​2.0-8.0 |
| B-Lymphocytes, cells x 10^9^/L |  |
| - < 2 days | 1.5-9.0 |
| - 2-6 days | 1.8-14.0 |
| - 7-28 days | 2.3-12.0 |
| - ​ 1-4 months | ​3.0-12.6 |
| - ​5-12 months | ​3.6-12.0 |
| - ​1-2 years | ​3.0-11.2 |
| - ​3-4 years | ​2.1-9.6 |
| - 5-6 years | ​1.5-8.3 |
| - 7-10 years | 1.5-6.5 |
| - ​11-17 years | 1.0-5.5 |
| B-Monocytes, cells x 10^9^/L |  |
| - < 2 days | ​0.5-2.5 |
| - ​ 2-6 days | ​0.3-3.2 |
| - 7-28 days | 0.2-2.0 |
| - ​ 1-4 months | 0.2-1.1 |
| - ​ 5-12 months | 0.2-1.0 |
| - ​ 1-2 years | ​0.2-1.0 |
| - ​3-4 years | 0.2-1.0 |
| - 5-6 years | 0.2-0.9 |
| - 7-10 years | ​0.2-0.8 |
| - ​ 11-17 years | ​0.1-0.8 |
| B-Eosinophils, cells x 10^9^/L |  |
| - < 2 days | ​0.0-1.0 |
| - ​ 2-6 days | ​0.2-2.0 |
| - 7-28 days | 0.1-1.2 |
| - ​ 1-4 months | ​0.0-0.7 |
| - ​ 5-12 months | 0.0-0.6 |
| - ​ 1-2 years | ​0.0-0.6 |
| - ​3-4 years | ​0.0-0.6 |
| - 5-6 years | 0.0-0.6 |
| - 7-10 years | ​0.0-0.5 |
| - ​ 11-17 years | 0.0-0.5 |
| B-Basophils, cells x 10^9^/L |  |
| - < 2 days | 0.0-0.1 |
| - ​ 2-6 days | ​0.0-0.1 |
| - 7-28 days | 0.0-0.1 |
| - ​ 1-4 months | ​0.0-0.1 |
| - ​ 5-12 months | ​0.0-0.1 |
| - ​ 1-2 years | ​0.0-0.1 |
| - ​3-4 years | ​0.0-0.1 |
| - 5-6 years | 0.0-0.1 |
| - 7-10 years | ​0.0-0.1 |
| - ​ 11-17 years | ​0.0-0.1 |
| B-Platelet count, cells x 10^9^/L |  |
| - <21 days | ​85-475 |
| - 21-28 days | ​80-600 |
| - 1-3 months | ​160-600 |
| - 4-12 months | 130-500 |
| - 1-17 years | 150-400 |
| P-C-reactive protein (CRP), mg/L | <3 |
| B-Erythrocyte sedimentation rate (ESR), mm/h |  |
| - Females | <20 |
| - Males | <10 |
| P-Albumin, g/L |  |
| - <15 days | ​28-41 |
| - 15 days- 26 weeks | ​25-46 |
| - ​27 weeks- <1 years | ​30-46 |
| - ​1-7 years | 35-45 |
| - 8-14 years | ​37-47 |
| - ​15-17 years | 36-49 |
| P/S-IgM, g/L |  |
| - <15 days | ​0.10-0.40 |
| - ​​15 days-2 months | ​0.10-0.70 |
| - ​3 months-<1 years | ​0.20-0.90 |
| - ​3 months-<1 years | ​0.50-1.70 |
| P/S-IgG, g/L |  |
| - <15 days | ​3.20-14.0 |
| - ​15 days-<1 years | 1.10-7.00 |
| - ​1-3 years | ​3.20-11.5 |
| - ​4-9 years | 5.40-13.6 |
| - ​10-17 years | ​6.60-15.3 |
| P-Creatinine, micromole/L |  |
| - ​​≤ 15 days | 77 |
| - ​15 days-2 months | 60 |
| - ​2 months-2 years | 41 |
| - 3 years | 44 |
| - 4 years | 47 |
| - 5 years | 50 |
| - 6 years | 53 |
| - 7 years | 56 |
| - 8 years | 59 |
| - 9 years | 62 |
| - 10 years | 65 |
| - 11 years | 68 |
| - 12 years | 71 |
| - 13 years, females | 74 |
| - 13 years, males | 84 |
| - 14 years, females | 77 |
| - 14 years, males | 87 |
| - 15 years, females | 80 |
| - 15 years, males | 90 |
| - 16 years, females | 83 |
| - 16 years, males | 93 |
| - 17 years, females | 86 |
| - 17 years, males | 96 |
| P-Aspartateaminotransferase (AST), microkat/L |  |
| - <0.5 years | ​<1.4 |
| - 0.5-1 years | ​<1.1 |
| - 2-17 years | <0.78 |
| P-Alanineaminotransferase (ALT), microkat/L |  |
| - ​<1 years | ​<0.85 |
| - 1-5 years | <0.50 |
| - 6-17 years | <0.52 |
| P-Gamma-glutamyltransferase (y-GT), mikrokat/L |  |
| - < 8 years | <0.27 |
| - 8-12 years | <0.43 |
| - 13-17 years | <0.60 |
| P-Bilirubin, micromole/L |  |
| - < 2 days | <100 |
| - 2-6 days | <200 |
| - 7-20 days | <100 |
| - 21-29 days | <50 |
| - 1-5 months | <22 |
| - 6 months-5 years | <8 |
| - 6-10 years | <18 |
| - 11-17 years | <24 |
| P-Lactatedehydrogenase (LDH), microkat/L |  |
| - 0-6 months | ≤10.0 |
| - 6 months-12 years | ​2.2-5.3 |
| - 13-17 years | ≤4.5 |

**Supplementary table 4.** Clinical presentation related to splenomegaly only in *Plasmodium* infected adults and children

|  | **All** | | | |  | **Adults, ≥18 years** | | | |  | **Children, <18 years** | |
| --- | --- | --- | --- | --- | --- | --- | --- | --- | --- | --- | --- | --- |
|  | **With splenomegaly**  (N=15) | **Without splenomegaly**  (N=44) |  | **p-value^a^** |  | **With splenomegaly** (N=11) | **Without splenomegaly** (N=18) |  | **p-value^a^** |  | **With splenomegaly** (N=4) | **Without  splenomegaly** (N=26) |
| **Malaria diagnostics, n/N (%)** |  |  |  |  |  |  |  |  |  |  |  |  |
| PCR-positive^b^ | 15/15 (100) | 44/44 (100) |  | - |  | 11/11 (100) | 18/18 (100) |  | - |  | 4/4 (100) | 26/26 (100) |
| RDT-positive^b^ | 6/15 (40.0) | 12/40 (30.0) |  | 0.529 |  | 3/11 (27.3) | 2/15 (13.3) |  | 0.620 |  | 3/4 (75.0) | 10/25 (40.0) |
| Microscopy-positive^c^ | 4/13 (30.8) | 12/28 (42.9) |  | 0.513 |  | 2/9 (22.2) | 4/12 (33.3) |  | 0.659 |  | 2/4 (50.0) | 8/16 (50.0) |
| ***Plasmodium* species^d^, n/N (%)** |  |  |  |  |  |  |  |  |  |  |  |  |
| *P. falciparum* | 9/15 (60.0) | 20/44 (45.5) |  | 0.382 |  | 7/11 (63.6) | 12/18 (66.7) |  | 1.000 |  | 2/4 (50.0) | 8/26 (30.8) |
| *P. ovale* | 1/15 (6.7) | 13/44 (29.5) |  | 0.090 |  | 0/11 (0) | 3/18 (16.7) |  | 0.269 |  | 1/4 (25.0) | 10/26 (38.5) |
| *P. malariae* | 3/15 (20.0) | 5/44 (11.4) |  | 0.407 |  | 2/11 (18.2) | 1/18 (5.6) |  | 0.539 |  | 1/4 (25.0) | 4/26.0 (15.4) |
| Mixed *Plasmodium* infection^e^ | 2/15 (13.3) | 6/44 (13.6) |  | 1.000 |  | 2/11 (18.2) | 2/18 (11.1) |  | 0.622 |  | 0/4 (0) | 4/26 (15.4) |
| **Days in Sweden (median (range)** | 221 (24-371) | 63 (7-449) |  | 0.164 |  | 158 (24-371) | 91 (9-449) |  | 0.921 |  | 275 (42-371) | 46 (7-449) |
| **Reported complaints^f^, n/N (%)** |  |  |  |  |  |  |  |  |  |  |  |  |
| Fever | 1/15 (6.7) | 7/44 (15.9) |  | 0.666 |  | 1/11 (9.1) | 3/18 (16.7) |  | 1.000 |  | 0/4 (0) | 4/26 (15.4) |
| Chills | 1/15 (6.7) | 4/44 (9.1) |  | 1.000 |  | 0/11 (0) | 4/18 (22.2) |  | 0.268 |  | 1/4 (25.0) | 0/26 (0) |
| Headache | 1/15 (6.7) | 8/44 (18.2) |  | 0.424 |  | 1/11 (9.1) | 5/18 (27.8) |  | 0.362 |  | 0/4 (0) | 3/26 (11.5) |
| Body ache | 0/15 (0) | 3/44 (6.8) | - | 0.564 |  | 0/11 (0) | 3/18 (16.7) |  | 0.269 |  | 0/4 (0) | 0/26 (0) |
| Gastrointestinal complaints | 3/15 (20.0) | 13/44 (29.5) |  | 0.738 |  | 2/11 (18.2) | 8/18 (44.4) |  | 0.234 |  | 1/4 (25.0) | 5/26 (19.2) |
| Cough | 1/15 (6.7) | 1/44 (2.3) |  | 0.447 |  | 1/11 (9.1) | 0/18 (0) |  | 0.379 |  | 0/4 (0) | 1/26 (3.8) |
| **Laboratory findings, n/N (%)** |  |  |  |  |  |  |  |  |  |  |  |  |
| Anemia | 3/15 (20.0) | 6/36 (16.7) |  | 1.000 |  | 3/11 (27.3) | 5/18 (27.8) |  | 1.000 |  | 0/4 (0) | 1/18 (5.6) |
| Leukopenia^g^ | 4/15 (26.7) | 9/36 (25.0) |  | 1.000 |  | 3/11 (27.3) | 1/18 (5.6) |  | 0.139 |  | 1/4 (25.0) | 8/18 (44.4) |
| Neutropenia | 8/12 (66.7) | 11/31 (35.5) |  | 0.091 |  | 6/10 (60.0) | 2/18 (11.1) |  | 0.011 |  | 2/2 (100) | 9/13 (69.2) |
| Eosinophilia | 1/12 (8.3) | 3/32 (9.4) |  | 1.000 |  | 1/10 (10.0) | 3/18 (16.7) |  | 1.000 |  | 0/2 (0) | 0/14 (0) |
| Thrombocytopenia | 4/15 (26.7) | 2/35 (5.7) |  | 0.058 |  | 4/11 (36.4) | 1/17 (5.9) |  | 0.062 |  | 0/4 (0) | 1/18 (5.6) |
| Elevated ESR | 7/9 (77.8) | 10/19 (52.6) |  | 0.250 |  | 6/8 (75.0) | 8/17 (47.1) |  | 0.234 |  | 1/1 (100.0) | 2/2 (100) |
| Hypoalbuminemia | 2/8 (25.0) | 7/22 (31.8) |  | 1.000 |  | 2/8 (25.0) | 5/17 (29.4) |  | 1.000 |  | 0/0 (-) | 2/5 (40.0) |
| Elevated IgM | 9/15 (60.0) | 7/38 (18.4) |  | 0.006 |  | 7/11 (63.6) | 5/16 (31.3) |  | 0.130 |  | 2/4 (50.0) | 2/22 (9.1) |
| Elevated IgG | 13/15 (86.7) | 30/38 (78.9) |  | 0.705 |  | 11/11 (100) | 14/16 (87.5) |  | 0.499 |  | 2/4 (50.0) | 16/22 (72.7) |
| Elevated LDH | 8/10 (80.0) | 9/18 (50.0) |  | 0.226 |  | 8/9 (88.9) | 9/17 (52.9) |  | 0.098 |  | 0/1 (0) | 0/1 (0) |
| **Co-infections, n/N (%)** |  |  |  |  |  |  |  |  |  |  |  |  |
| Schistosomiasis | 6/15 (40.0) | 14/41 (34.1) |  | 0.758 |  | 5/11 (45.5) | 10/18 (55.6) |  | 0.710 |  | 1/4 (25.0) | 4/23 (17.4) |
| Strongyloidiasis | 4/15 (26.7) | 8/41 (19.5) |  | 0.715 |  | 3/11 (27.3) | 4/18 (22.2) |  | 1.000 |  | 1/4 (25.0) | 4/23 (17.4) |
| Latent tuberculosis | 2/14 (14.3) | 9/40 (22.5) |  | 0.708 |  | 2/10 (20.0) | 8/16 (50.0) |  | 0.218 |  | 0/4 (0) | 1/24 (4.2) |
| HIV | 0/13 (0) | 0/42 (0) |  | - |  | 0/9 (0) | 0/17 (0) |  | - |  | 0/4 (0) | 0/25 (0) |
| Hepatitis B | 0/13 (0) | 1/41 (2.4) |  | 1.000 |  | 0/9 (0) | 1/16 (6.3) |  | 1.000 |  | 0/4 (0) | 0/25 (0) |
| Hepatitis C | 0/13 (0) | 0/42 (0) |  | - |  | 0/9 (0) | 0/17 (0) |  | - |  | 0/4 (0) | 0/25 (0) |

Variables presented as n/N (%), where N equals number of study participants with available data for each variable. Odds ratios as well as p-values in children were not determined, since the small number of children with splenomegaly and the amount of missing data.

Abbreviations: RDT – rapid diagnostic test, ESR – erythrocyte sedimentation rate, LDH – lactate dehydrogenase

^a^ Fisher’s exact test; Mann-Whitney U; PCR-positive adults with and without splenomegaly compared

^b^ Research laboratory analysis

^c^ Clinical laboratory analysis

^d^ Determined by PCR

^e^  2 *P. falciparum*+*P. ovale*+P*. malariae*, 4 *P. falciparum*+*P. ovale*, 2 *P. falciparum*+*P. malariae*,

1 *P. ovale*+*P. malariae*

^f^ From arrival in Sweden until first clinical assessment for parasitic infection identified through screening

^g^ No leukocytosis observed

**Supplementary Table 5.** Study participants with *Plasmodium* infection and splenomegaly fulfilling HMS criteria

|  | ***Plasmodium* infection and splenomegaly**  (N=15)  **n/N (%)** |
| --- | --- |
| **HMS major criteria *(Fakunle 1981)*** |  |
| Gross splenomegaly ^a^ | 2/15 (13.3) |
| Immunity to malaria ^b, c^ | 15/15 (100) |
| Serum IgM at least two SD above local mean^d^ | 9/15 (60.0) |
| Clinical and immunological response to antimalarials | 11/11 (100) |
|  |  |
| Study participants fulfilling all major HMS criteria | 2/15 (13.3) |
|  |  |
| **Early HMS criteria *(Bisoffi 2015)*** |  |
| Anti-malarial antibody titre (IFAT-Biomérieux) > 1/160 ^b, c^ | 15/15 (100) |
| *AND* |  |
| Splenomegaly (echographic longitudinal diameter ≥ 12 cm or palpable lower pole of the spleen) | 15/15 (100) |
| *AND/OR* |  |
| High IgM level (≥2.5 g/L) | 7/15 (46.7) |
| No other identified causes of splenomegaly or of raised IgM | 15/15 (100) |
|  |  |
| Study participants fulfilling early HMS criteria | 15/15 (100) |

Condensed version of HMS and eHMS criteria, for complete criteria, see Supplementary Table 1.

Abbreviations: HMS – Hyper-reactive malarial splenomegaly

^a^ Here defined also defined as spleen length of ≥16 cm determined through radiological examination as in Bisoffi,

Leoni, Angheben et al, Malar J, 2016 (13)

^b^ Here interpreted as positive *P. falciparum* crude schizont extract serology

^c^ One participant had initially negative malaria (*P. falciparum*) serology. This sample was reanalyzed two times and was

then serology positive, thus this individual has been interpreted as having positive malaria serology results.

^d^ >2.10g/L according to local reference ranges at the Karolinska University Hospital, Stockholm

| 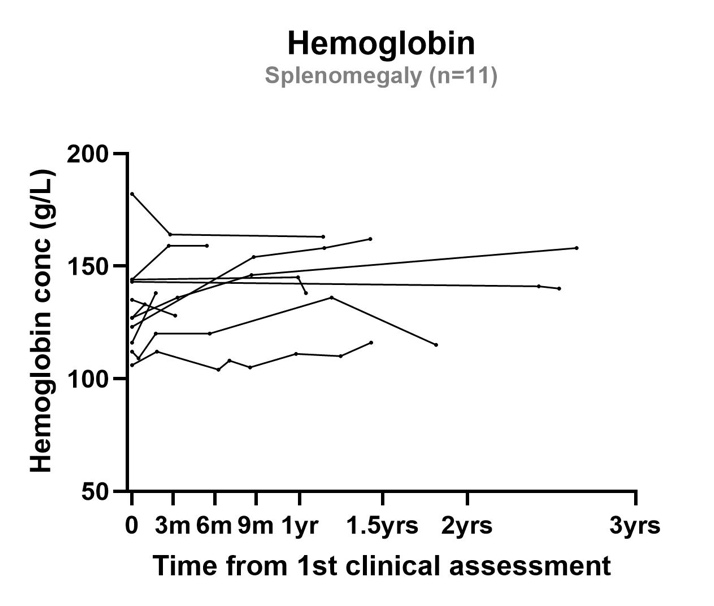 | 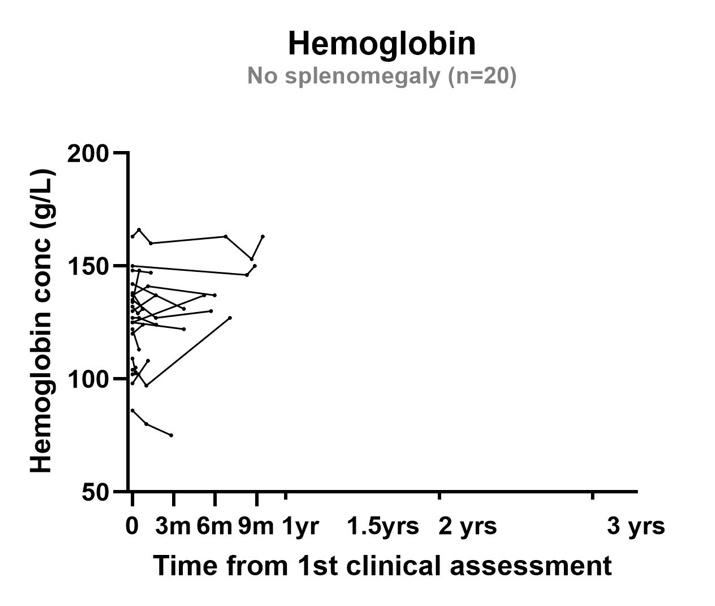 |
| --- | --- |
| 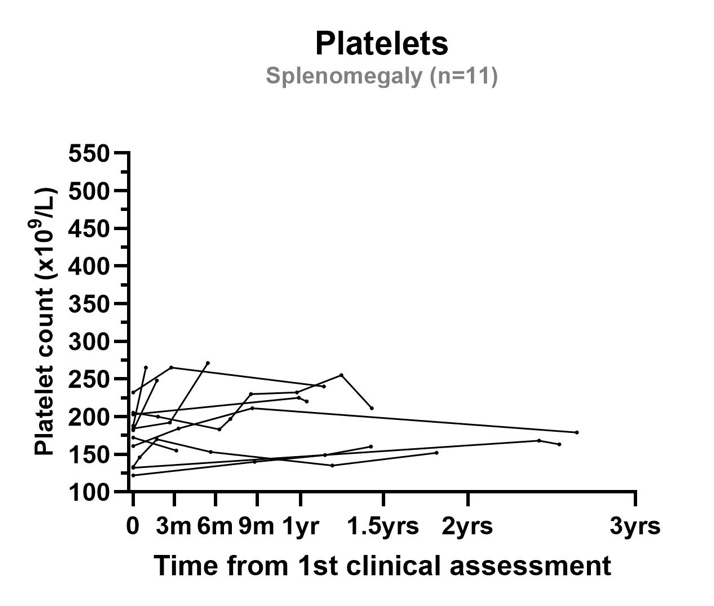 | 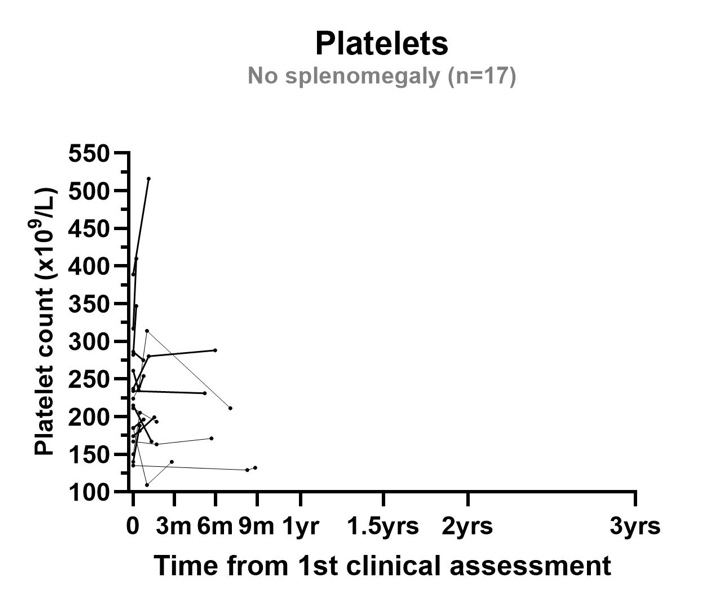 |
| 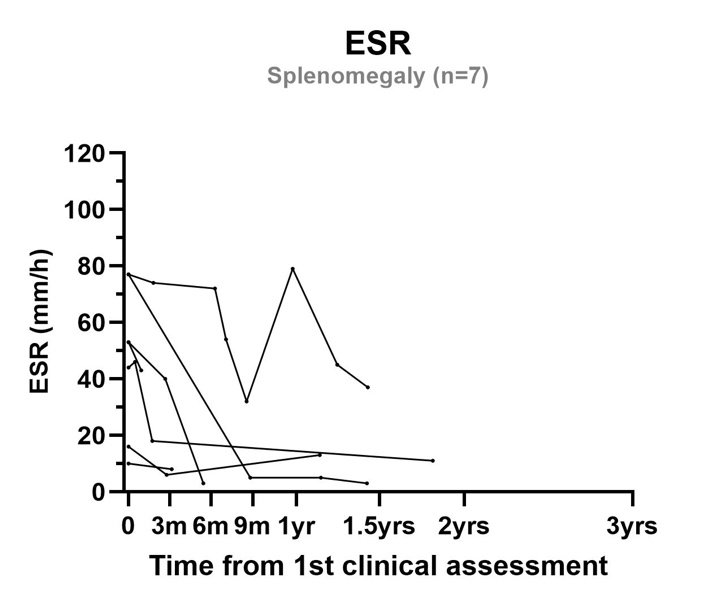 | 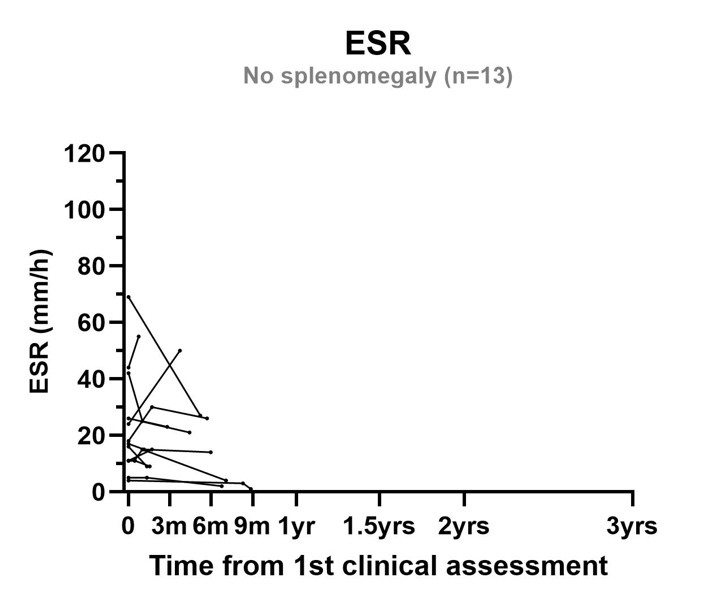 |
| 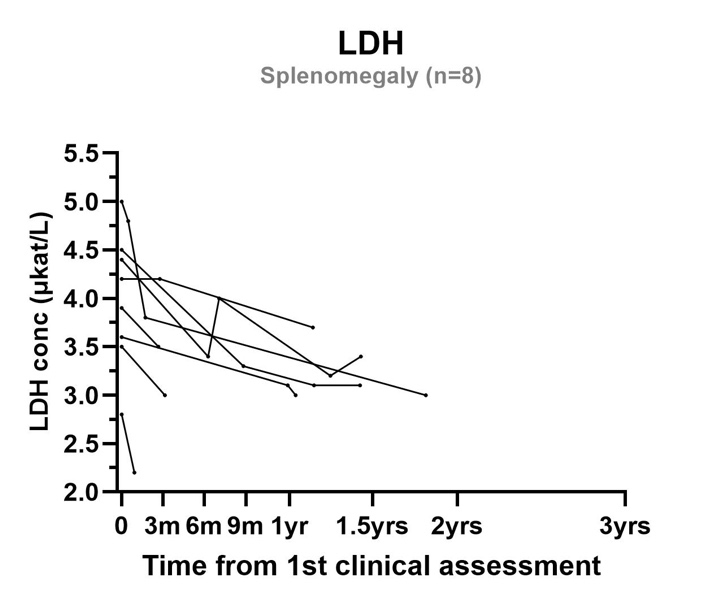 | 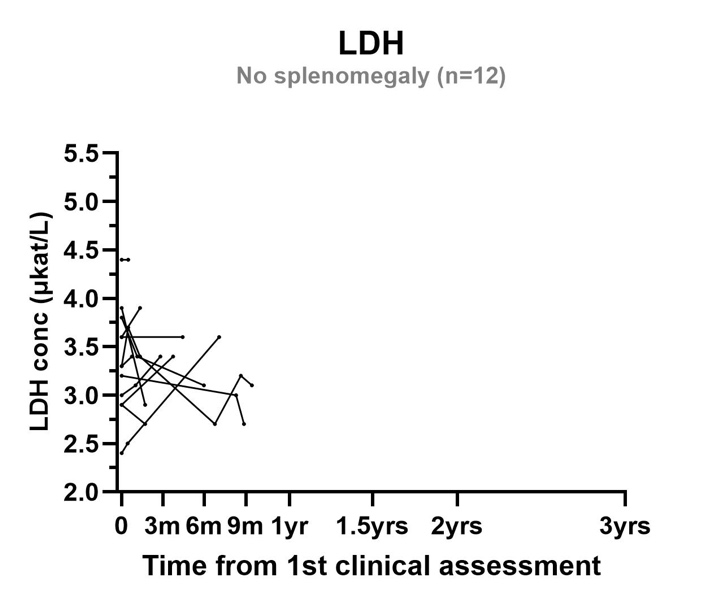 |

**Supplementary figure 1.** Selected laboratory parameters in study participants with *Plasmodium* infection over time divided by splenomegaly status (individual). Time from first clinical assessment when antimalarial treatment was provided.

| 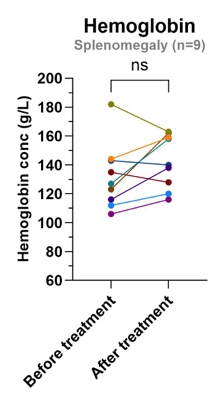 | 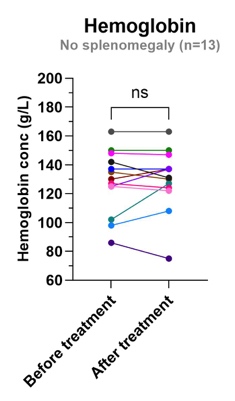 | 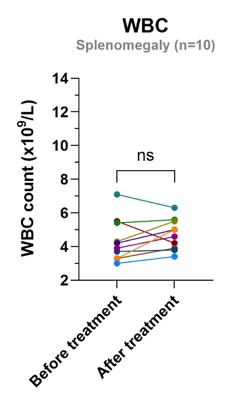 | 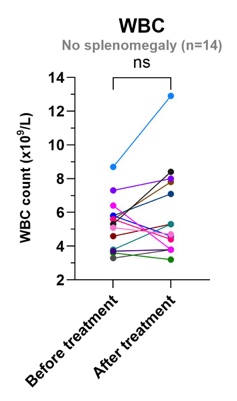 | 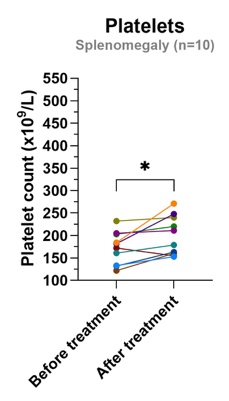 | 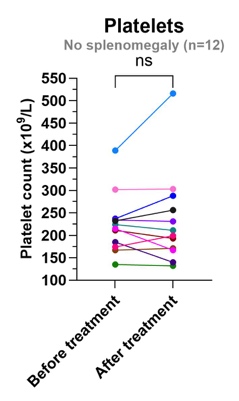 |
| --- | --- | --- | --- | --- | --- |
| 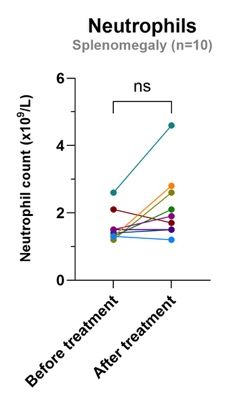 | 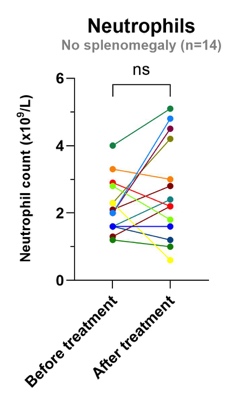 | 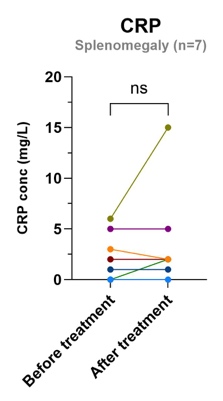 | 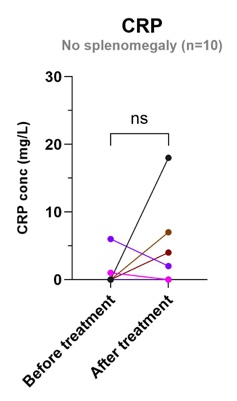 | 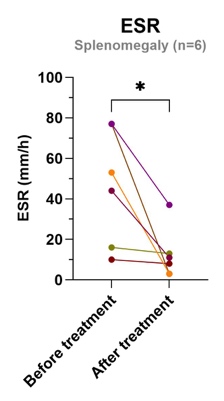 | 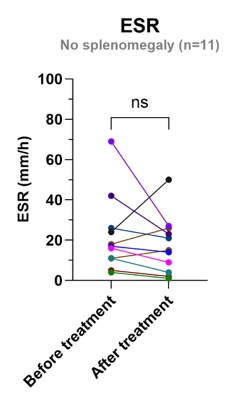 |
| 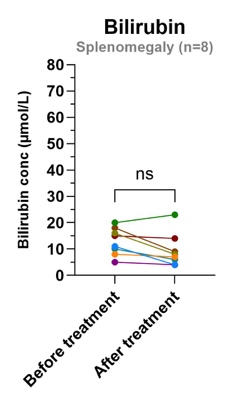 | 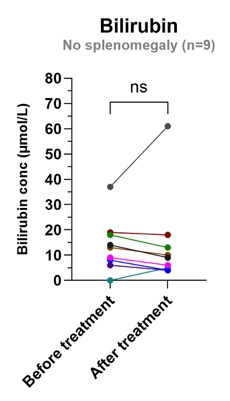 | 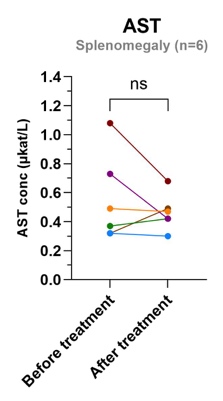 | 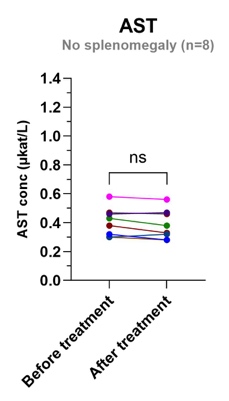 | 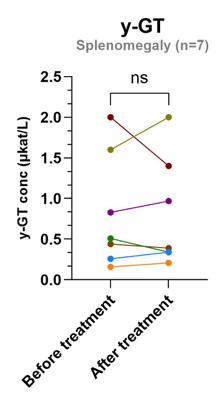 | 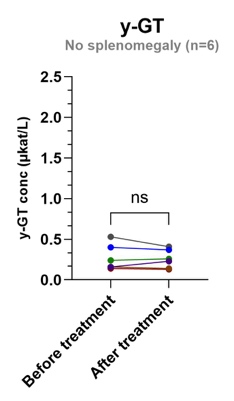 |
| 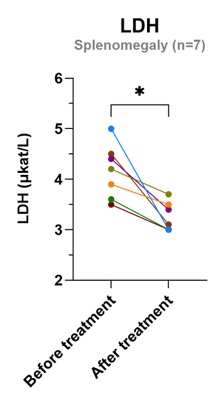 | 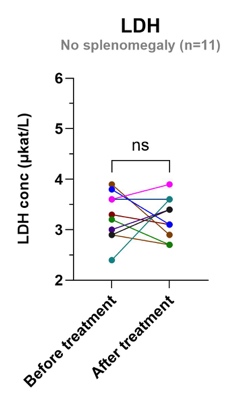 | 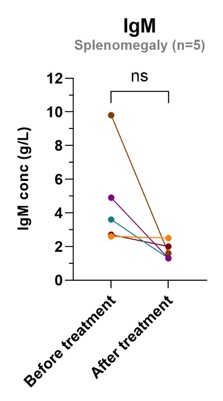 | 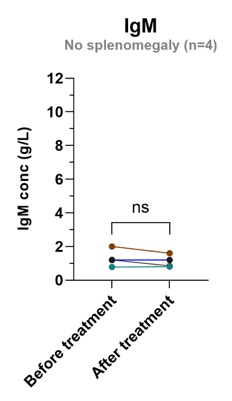 | 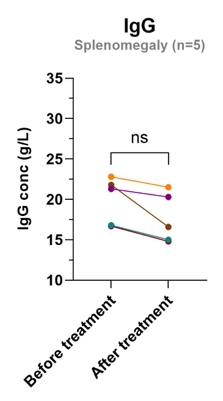 | 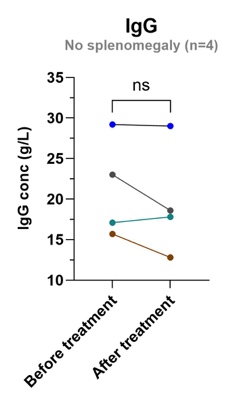 |

**Supplementary figure 2.** Laboratory findings in adults and children before and after antimalarial treatment in *Plasmodium* infected with and without splenomegaly. Before treatment represents laboratory findings from the initial clinical visit for evaluation of *Plasmodium* infection found through screening. After treatment represents laboratory findings at the most recent clinical follow-up visit with available test results after receiving antimalarial treatment. Follow-up visits <1 month from baseline were excluded. Statistical analysis with Wilcoxon matched-pairs signed rank test, ns p≥0.05, * p<0.05, ** p<0.01,*** p<0.001, **** p<0.0001.
